# Supplementary material for: A Double-Blind, Randomized, Placebo-Controlled Trial of the Effect of 1-Kestose on Defecation Habits in Constipated Kindergarten Children: A Pilot Study
Source: Nutrients. 2023 Jul 24;15(14):3276. doi: 10.3390/nu15143276 (PMC10386190; doi:10.3390/nu15143276)
Supplement: Supplementary file 1 [file nutrients-15-03276-s001.zip › Supplemental Table S1_np.pdf]

Supplemental Table S1. Participant characteristics

	Kestose (n=11)	Maltose (n=12)	p
Age at entry, years, median (25th–75th %)	5 (4.5-5.5)	5 (5-6)	0.36
Sex, boy/girl	6/5	9/3	0.30
Taking pre/probiotics	5	6	0.48
Taking laxatives	2	1	0.83

Pre/probiotics: yogurt, lactic-fermented beverage, health foods (oligosaccharides, dietary fiber)
