# Supplementary figures and images for: A Double-Blind, Randomized, Placebo-Controlled Trial of the Effect of 1-Kestose on Defecation Habits in Constipated Kindergarten Children: A Pilot Study
Source: Nutrients. 2023 Jul 24;15(14):3276. doi: 10.3390/nu15143276 (PMC10386190; doi:10.3390/nu15143276)

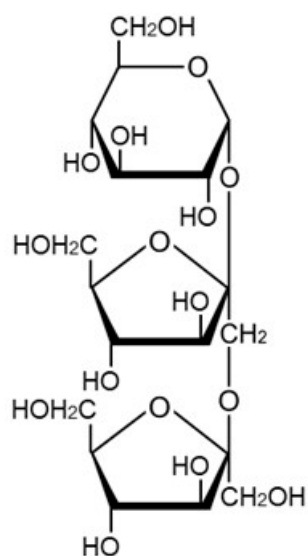

**Supplemental Figure S1.** 1-kestose  
 a fructose molecule connected to sucrose by a (1 → 2β)  
 glycosidic bond

Supplement: Supplementary file 1 [file nutrients-15-03276-s001.zip › Supplemental Figure S1_np.pdf]

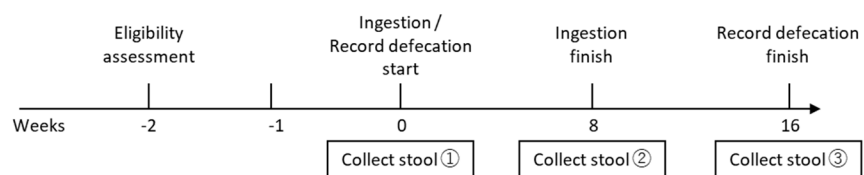

**Supplemental Figure S2.** Test schedule

Supplement: Supplementary file 1 [file nutrients-15-03276-s001.zip › Supplemental Figure S2_np.pdf]

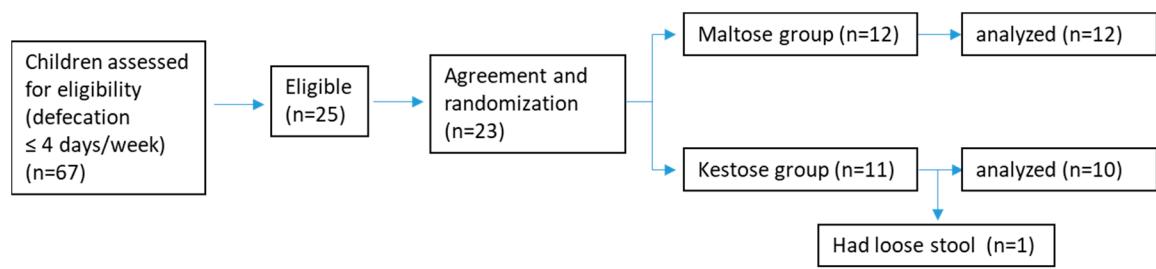

**Supplemental Figure S3** Flowchart of the study recruitment

Supplement: Supplementary file 1 [file nutrients-15-03276-s001.zip › Supplemental Figure S3_np.pdf]

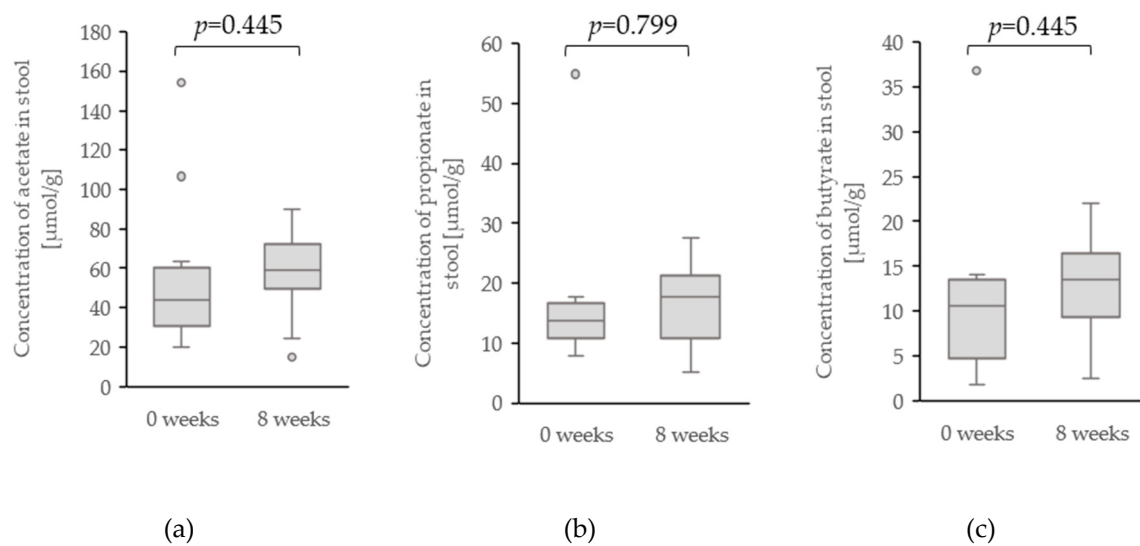

**Supplemental Figure S4.** SCFA concentration in stool. a) Acetate; b) propionate; c) butyrate

Supplement: Supplementary file 1 [file nutrients-15-03276-s001.zip › Supplemental Figure S4_np.pdf]
